# Quantitative real-time PCR assisted cell counting (qPACC) for epigenetic - based immune cell quantification in blood and tissue

**DOI:** 10.1186/s40425-015-0087-8

**Published:** 2015-11-17

**Authors:** Thomas Oliver Kleen, Jianda Yuan

**Affiliations:** Epiontis GmbH, Rudower Chaussee 29, 12489, Berlin Germany; Immune Monitoring Core, Ludwig Center for Cancer Immunotherapy, Memorial Sloan Kettering Cancer Center, 1275 York Ave, Box 386, New York, NY 10065 USA

**Keywords:** Immune monitoring, Epigenetic, Biomarker, Cell quantification, Cell-mediated immunity

## Abstract

A novel form of immune cell quantification in blood and tissue is described using epigenetic - based, quantitative real-time PCR assisted cell counting (qPACC). The methylation status of the chromatin structure of either actively expressed or silenced genes is the basis of the epigenetic-based cell identification and quantification technology. Discovery of cell type specific removal of methyl groups (demethylation) from the 5′-carbon of the cytosine base in the dinucleotide cytosine phosphate guanine permits precise and robust quantification of immune cells from only small amounts of human blood or tissue samples. These epigenetic biomarkers located on genomic DNA are stably associated with a cell type of interest.

## Description of the technology

All the different cell types that make up the human body generally share the exact same deoxyribonucleic acid (DNA). However, not all cells have the same function and identity. Epigenomics is providing an explanation through the study of the key functional components that regulate gene expression in a cell. Despite having the same DNA, sequence specific, epigenetic marks in each cell type activate certain gene sequences and silence others so that various progenitor cells can differentiate into specialized cell types like muscle, liver, bone or immune cells. These marks, like DNA methylation, occur without changing the primary DNA sequence but can be passed on as epigenetic imprint from cell to cell as they divide [1]. The NIH Roadmap Epigenomics Consortium performed an analysis of more than one hundred human reference epigenomes and profiled those for DNA methylation, DNA accessibility, histone modification patterns and RNA expression. They established global maps of regulatory elements and defined regulatory modules of coordinated activity as well as their likely activators and repressors, thereby explaining how cell-specific programs of gene expression are achieved and transcriptional and translational control is ensured. These novel data provide a valuable resource for understanding the role of epigenomic information in gene regulation, cellular differentiation and the relationships between cells and tissues [1].

**Fig. 1:**
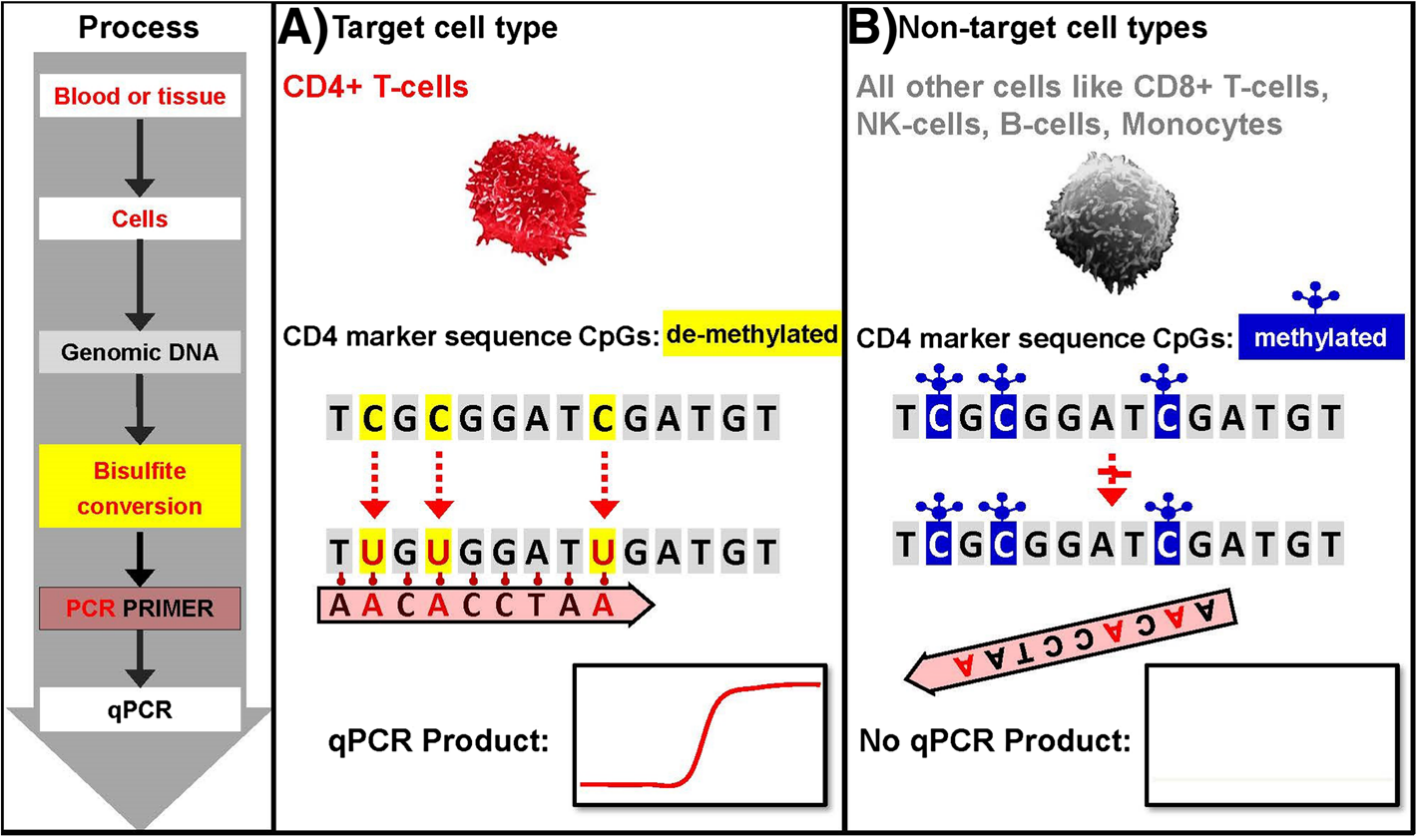
Assay principle – BSC treated genomic DNA specific qPCR. **a** Only demethylated cytosines in the target cell type (*i.e.* CD4+ T-cells) convert to uracil during bisulfite conversion (BSC). This allows BSC genomic DNA specific segregating primer and probes to bind and lead to a qPCR product. **b** Methylated cytosines in non - target cells do not change during BSC treatment of genomic DNA leading to a primer mismatch and no qPCR product

One of these epigenetic marks, the methylation status of the chromatin structure of either actively expressed or silenced genes, is the basis of the cell identification and quantification technology described here. Epigenetic differentiation is based on addition and removal of a methyl group to the 5’-carbon of the cytosine base and occurs exclusively in the dinucleotide cytosine phosphate guanine (CpG). DNA methylation is a non-random event and often associated with inactive gene expression, if the target CpGs are located in the proximity of coding regions. In contrast, demethylation of CpG in regulatory elements is commonly accompanied by activation of gene expression. Recent discovery of cell type specific epigenetic CpG demethylation markers permits precise and robust quantification of immune cells from only small amounts of human blood or tissue samples [2]. These epigenetic biomarkers located on genomic DNA are stably associated with a cell type of interest [3]. The first step, the epigenetic assay development phase, is only necessary once for every cell type or subtype for which an immune cell marker is being developed. Here, different cell types of interest suitable for assay development are identified by cell type and subtype-specific epigenetic marker regions during genome wide differential CpG demethylation analyses of highly purified cell populations. The initial purification is performed by cell sorting by flow cytometry (FACS) and based on consensus markers from the literature defining an immune cell type that an assay is being developed for. The demethylation status of every CpG position in DNA of a FACS purified cell type of interest is compared against a library of other reference cell types with known CpG methylation status. The regions that are selected for the development of a cell type specific epigenetic assay are based on specific DNA sequences with digitally differential bisulfite conversion (BSC) properties between the different cell types. This means the respective CpG dinucleotides must be fully demethylated in the cell type of interest and methylated in all other cell types. During BSC, unmethylated cytosines convert to uracil, but methylated cytosines do not change. Resulting determination of a cell type specific demethylation status in relevant loci is the basis for the development of segregating primer and probes. The cell quantitation methodology of any developed assay is based on quantitative real-time PCR (qPCR), targeting those differentially demethylated CpG marker regions in the genomic DNA after a bisulfite conversion step (see Fig. 1).

The cell type specific qPCR assays are designed such that only the demethylated DNA is amplified. This facilitates subsequent fast quantification of various leukocyte and other cell populations in any given DNA containing sample by simple qPCR.

## Type of data obtained/readout

The final read out of epigenetic differentiation assays is reported as percentage measured of cell type of interest of all other cells in a sample or, in addition, as percentage compared to other cell types measured in parallel. Representative examples of assays developed are based on complete demethylation of the Treg cell-specific demethylated region (TSDR) in regulatory T cells (Treg) [2, 3], the demethylated region in the intergenic region of CD3D/CD3G in T cells [4], or the demethylation within the CCR6 locus in CCR6-positive cells [5]. Various epigenetic immune cell biomarkers have been identified, characterized and validated for regulated studies to maintain intra-assay ≤ 15 % CV and inter-assay ≤ 20 % CV, including those for Treg, Th17, Tfh, CD4+, CD8+ and CD3+ T cells, B cells, Monocytes, NK cells and granulocytes. Methodology for data analysis and specific mathematical models have been published and it has been demonstrated that group differences in epigenetic data can be detected reliably [6].

## Limitations of the approach

It would be beneficial to identify more cell type specific epigenetic markers, including ones for additional subsets and differentiation stages of immune cells, especially activated and differentiated T cell populations known to play critical roles in tumor immunology and autoimmune diseases. Epigenetic differentiation of immune cells that are defined as effector versus memory T cells based on surface markers in flow, as well as activated cells versus exhausted T cells, would increase further the amount of actionable information derived with these assays.

## Types of samples needed and special issues pertaining to samples

The inherent stability of DNA and its methylation marks provides the distinctive advantage that epigenetic assays are non-susceptible to common stability and sample shipment logistics concerns of other comparable assays and technologies that often require intact, viable or functional cells from blood and tissue samples. This permits a significantly broader range of acceptable sample conditions that can be collected by clinical sites. Shipment is straightforward since whole blood and fresh/frozen solid tissue samples can be shipped on dry ice and FFPE can be used after shipment at room temperature. The currently available assays cover the major leukocyte types and can be evaluated in regulated, clinical studies requiring a total of 2 ml or less whole blood for all markers combined. The amount of sample required for such studies is being lowered by a factor of 20 to about 100 μl for all available assays combined in the near future. Frozen tissue material requires 250 μg to 1 mg tissue to measure twelve markers, less for fewer markers. The exact material needed is less defined compared to blood since the number of cells, and therefore the DNA content in tissue, varies significantly by weight or volume.

## Level of evidence

About 40 papers have been published describing and using this epigenetic differentiation based cell identification and quantification platform. Oncology has been an important area. The ratio of Treg to CD3 cells (immunoCRIT) measured with this technology has been shown as a potential marker for aggressiveness of solid tumors in colorectal, bronchial, mammary and ovarian cancers [7]. Other investigational fields include transplanation, type 1 diabetes, autoimmune hepatitis, dermatitis, as well as allergy and asthma. Common clinical trial applications of this technology are monitoring cell-mediated imunity (CMI) during immune-modulatory investigations for oncology and inflammatory diseases. Treg and CD3 cell monitoring in peripheral blood of MS patients in the SELECT study, a randomized, double-blind, placebo-controlled phase 2 clinical trial of the daclizumab high-yield process (DAC HYPE), is a recent example of an autoimmune, inflammatory disease setting that can be supported by this immune monitoring technology. It demonstrated that Treg phenotype and lineage stability can be maintained in the face of CD25 blockade [8]. Another example is measurements of CD3+, Treg (FoxP3) and Th17 cells in samples of a Phase 2 study of etrolizumab (EUCALYPTUS trial), an investigational drug for the treatment of ulcerative colitis (submitted for publication). Since the tests can be applied on both blood and tissue, they allow standardized measurements and comparison of circulating and tissue-infiltrating immune cells offering an alternative to flow cytometry for peripheral blood samples and immunohistochemistry (IHC) in solid tissues. The technology has significant differentiation points to flow cytometric-based methodologies, which are standard quantitative assays to monitor immunodynamics in blood. For example, the epigenetic based assay allows quick quantification and unambiguous differentiation of true Tregs from transiently Foxp3 expressing, activated effector T cells, which can be challenging and complex with flow cytometry [2, 9]. So far, there is a limited amount of data from clinical studies published. The release of additional clinical study data and future clinical studies are crucial to further validate the potential application of this novel technology for disease diagnosis and biomarker discovery for cancer immunotherapy.

## Abbreviations

BSC: Bisulfite conversion
CpG: Cytosine phosphate guanine
DNA: Deoxyribonucleic acid
FFPE: Formalin-fixed paraffin-embedded tissue
FACS: Flow cytometry
CMI: Cell-mediated immunity
immunoCRIT: Cellular ratio of immune tolerance
IHC: Immunohistochemistry
qPCR: quantitative real-time PCR
qPACC: quantitative real-time PCR assisted cell counting
TSDR: Treg cell-specific demethylated region
